# Natural Radioactivity Content and Radon Exhalation Rate Assessment for Building Materials from the Archaeological Park of Tindari, Sicily, Southern Italy: A Case Study

**DOI:** 10.3390/ijerph22030379

**Published:** 2025-03-05

**Authors:** Francesco Caridi, Giuseppe Paladini, Francesco Gregorio, Stefania Lanza, Gabriele Lando, Marco Sfacteria, Stefania Tuccinardi, Marta Venuti, Paola Cardiano, Domenico Majolino, Valentina Venuti

**Affiliations:** 1 Dipartimento di Scienze Matematiche e Informatiche, Scienze Fisiche e Scienze della Terra, Università degli Studi di Messina, 98166 Messina, Italy; gpaladini@unime.it (G.P.); francesco.gregorio1@studenti.unime.it (F.G.); stefania.lanza@unime.it (S.L.); dmajolino@unime.it (D.M.); vvenuti@unime.it (V.V.); 2 Dipartimento di Scienze Chimiche, Biologiche, Farmaceutiche e Ambientali, Università degli Studi di Messina, 98166 Messina, Italy; gabriele.lando@unime.it (G.L.); paola.cardiano@unime.it (P.C.); 3 Dipartimento di Civiltà Antiche e Moderne, Università degli Studi di Messina, 98100 Messina, Italy; marco.sfacteria@unime.it (M.S.); stefania.tuccinardi@unime.it (S.T.); marta.venuti@unime.it (M.V.)

**Keywords:** limestone, sandstone, radioactivity, radiological risk, radon exhalation

## Abstract

This paper presents a case study of the natural radioactivity level and radon exhalation in limestone and sandstone rocks from the archaeological park of Tindari, located in Sicily, southern Italy. These rocks were representative of natural stones utilised as building materials in the studied area. The activity concentrations of ^226^Ra, ^232^Th, and ^40^K were assessed using high purity germanium (HPGe) gamma-ray spectrometry. Subsequently, the absorbed gamma dose rate (D), annual effective dose equivalent (AEDE), activity concentration index (ACI), and alpha index (I_α_) were quantified to evaluate potential radiological health risks associated with radiation exposure from the analysed rocks. Finally, E-PERM electret ion chamber measurements were conducted to accurately quantify the radon exhalation rate from the investigated samples. The results obtained in this case study provide a foundation for further research into the background radioactivity levels in natural stones employed as building materials.

## 1. Introduction

It is well-established that humans are continuously exposed to radiation from natural sources throughout their lives [1]. This exposure arises from various naturally occurring radioactive materials (NORMs) present in the Earth’s environment including rocks, soil, water, and atmosphere [2,3,4]. Both humans and other living organisms inevitably accumulate background radiation through daily interactions with these sources [5]. Among the NORMs, ^226^Ra, ^232^Th, and ^40^K, originating from the natural decay of uranium, thorium, and potassium in the Earth’s crust [6,7], respectively, are the primary contributors to background radiation [8,9]. Radioactivity levels vary by location due to differences in geographical conditions and geological structures [10,11]. For instance, regions containing uranium- or thorium-rich minerals tend to exhibit higher background radiation levels, while areas with distinct geological characteristics may have lower natural radioactivity [12].

Based on the aforementioned considerations, investigating the natural radioactivity content of rocks, especially if employed as building materials, is essential for assessing the radiological risk posed by ionising radiation exposure [13,14]. In fact, as reported in the literature [15], when human beings are exposed to ^234^U, ^235^U, ^238^U, ^226^Ra, ^228^Ra, and ^232^Th over a long period of time—whether by ingestion or inhalation—they can suffer various health effects [16]. Building materials can be radioactive for a number of reasons, mainly because the raw materials contain elevated concentrations of natural radioactivity [17]. It is therefore essential to continuously monitor the specific activity of radionuclides in construction materials. One of the approaches used to reduce the external dose is the selection of materials with low radionuclide activity. The assessment of doses due to radioisotopes in construction materials is important from the point of view of radiological safety. The high activity of natural radionuclides in construction materials can lead to higher dose rates in indoor environments, especially when products from different manufacturers are used in production [17].

Additionally, chronic exposure to radon gas (^222^Rn) has been linked to an increased risk of lung cancer [18]. The World Health Organisation (WHO) identifies radon and its progeny as the second leading cause of lung cancer in the general population, following smoking [19,20]. Consequently, monitoring the radon levels and its decay products is crucial for assessing radiological risk, which is heightened in smokers and primarily attributed to the ^214^Po and ^218^Po radioisotopes [21]. The exhalation of ^222^Rn from rock surfaces is strongly influenced by petrographic and petrophysical properties such as microfractures, particle size, mineral structure, degree of alteration, and contact surfaces between constituents. While ^238^U/^226^Ra activity measurements provide valuable insights into radon release potential, they do not fully capture the real-world exhalation dynamics [22]. Therefore, real-time investigations of ^222^Rn exhalation rates are necessary to accurately evaluate the radiological risk associated with natural stone materials [23].

It is worth noting that, since 1989, the European Union (EU) has implemented a series of regulations and directives with the aim of controlling the radiation emissions from building materials, with a particular focus on their potential impact on human health. Notably, in 1999, the European Commission (EC) unveiled the ALARA (As Low As Reasonably Achievable) principle, establishing the objective of radiation control in building materials as the limitation of exposure to radiation from substances with high levels of natural radioactivity [24]. In more recent years, the EU has considered the radiological risk to public health in the European Directive 2013/59 EURATOM [25]. In particular, this directive underscores the significance of the investigation into all potential sources of indoor radon including soil, building materials, water, and natural gas supplied by domestic systems. The overarching objective of this directive is to enhance the biocompatibility of homes from a radiological perspective [26].

On the basis of the aforementioned, in this study, high purity germanium (HPGe) gamma-ray spectrometry and E-PERM electret ion chambers were employed to investigate, for the first time, the natural radioactivity levels and radon exhalation in limestone and sandstone rocks from the archaeological park of Tindari, Sicily region, southern Italy, which was used as a case study [27].

In particular, the residential buildings of the ancient city of Tindari were constructed using a technique that incorporated stone fragments and bricks set in mortar. Among the stone fragments, a compact grey limestone—likely locally quarried—was predominantly used. The hill on which the ancient city stands is composed entirely of grey limestone, which remains exposed and visible in several locations within the archaeological park [28]. In contrast, sandstone was used in the construction of the Greek-Roman Theatre of Tindari, one of Sicily’s most significant archaeological monuments, located on the slope of Capo Tindari hill in the southeastern section of the ancient city, approximately 50–60 m from the still-preserved city walls [29,30].

Finally, the absorbed gamma dose rate (D), annual effective dose equivalent (AEDE), activity concentration index (ACI), and alpha index (I_α_) radiological parameters were calculated to assess any possible hazards associated with ionising radiation exposure from the investigated limestone and sandstone rocks.

## 2. Geological Setting

The Promontory of Capo Tindari, situated along the Tyrrhenian margin of the Peloritani Belt in northeastern Sicily, forms part of a tectonically complex region. The area is composed of a set of south-verging tectonic units that were emplaced during Oligocene times [31]. These units consist of various tectonic napes comprising Hercynian basement rocks overlain by Meso-Cenozoic sedimentary successions. Overlying these older formations are Holocene deposits of marine and continental origin. These include terraced marine deposits, colluvial deposits at the base of scarps and slopes, fluvial sediments in valley floors, beach deposits, and the noteworthy Marinello lagoons situated at the foot of the Capo Tindari promontory. These lagoons represent dynamic geomorphological features influenced by coastal processes and sediment transport [32]. The structural evolution of the region has been predominantly governed by the Tindari Fault System, an active fault network trending NNW. This system connects the central Aeolian Islands with the Ionian coast of Sicily, northeast of Mount Etna. Fault activity in the area is attributed primarily to the Middle-Late Pleistocene, with evidence derived from faulted Pleistocene marine terraces and tectonically modified Quaternary deposits [33].

## 3. Materials and Methods

### 3.1. Sampling

The investigated samples consisted of limestone and sandstone rocks, both collected within the archaeological park of Tindari, specifically at the site with GPS coordinates 38°08′37.8″ N and 15°02′34.7″ E, which are representative of the entire archaeological site in terms of the type of analysed natural stones.

In the laboratory, five aliquots of each rock type were prepared by cutting larger stone pieces by means of a circular saw. Each aliquot was shaped into a cube of approximately 5 cm.

**Figure 1:**
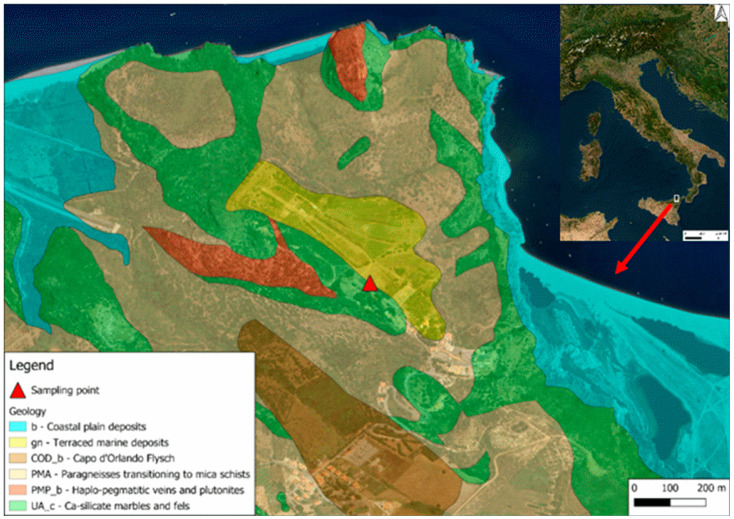
Geological map of the investigated area along with the position of the sampling site indicated.

The geological map of the investigated area, together with the position of the sampling site, is shown in Figure 1. The sampling point is located on a terraced marine deposit, made up of yellow ochre sands, sometimes gravelly, and gravels with rounded and heterometric pebbles, mostly crystalline. The lower boundary surface was modelled on the crystalline base made up of considerable thicknesses of marble. The age of this terrace is Middle-Upper Pleistocene.

### 3.2. High Purity Germanium (HPGe) Gamma Spectrometry

For HPGe analysis, each aliquot was treated at the laboratory as reported in [34]. A live time of 70,000 s was used, and the activity concentrations were determined as follows:

(i) ^226^Ra: Based on the 295.21 and 351.92 keV (^214^Pb) and 1120.29 keV (^214^Bi) gamma-ray lines;
(ii) ^232^Th: Using the 911.21 and 968.97 keV (^228^Ac) gamma-ray lines;
(iii) ^40^K: Evaluated from its 1460.8 keV gamma-ray line [35,36].

The gamma spectrometry analysis was carried out through an electrically cooled Ortec HPGe detector (Ametek Ortec, Oak Ridge, TN, USA), housed within lead wells to minimise the background radiation [37] (i.e., a direct biased semiconductor with 1.85 keV FWHM resolution, 40% relative efficiency, and 64:1 peak to Compton ratio). The Eckert and Zigler Nuclitec GmgH (Eckert & Ziegler, Braunschweig, Germany) traceable multinuclide radioactive standard, number BC4464, covering the energy range 59.54–1836.09 keV, was employed to perform the efficiency and energy calibrations [38]. The exact geometry of the sample was replicated by this calibration standard in an epoxy resin matrix water equivalent. Data collection and analysis were performed using Ortec Gamma Vision software version 8 [39].

The activity concentration (Bq kg^−1^ dry weight, d.w.) of each radioisotope was quantified according to:

(1) $$C\left(Bq\;{kg}^{-1}\;d.w.\right)=\frac{N_{E}}{\varepsilon_{E}t\gamma_{d}M}$$

where N_E_, ε_E_, γ_d_, M, and t account for the net area, efficiency for energy E, decay probability of the gamma photon, dry mass of the sample (kg), and acquisition time (s), respectively.

For the assessment of the combined standard measurement uncertainty at coverage factor k = 2, the counting statistics, nuclear data library, calibration efficiency, sample quantity, and self-absorption correction were considered [40]. For the detection limit evaluation, the RISO method was applied [40].

Moreover, in order to take self-absorption into account (i.e., the phenomenon according to which there is an absorption of radiation by the matrix itself that emits it), it is important to underline that (i) for photons of an energy greater than 100 keV, self-absorption depends almost exclusively on the density of the sample; and (ii) for photons of an energy less than 100 keV, it is also necessary to consider the effect of the chemical composition of the analysed matrix. Therefore, the bulk density of the sample must be assessed and verified so that it is within the range of acceptability defined by the laboratory [40]. In cases where it is also necessary to consider the effect of the chemical composition of the sample, the same should be estimated at least approximately (also using the literature data) and verified that it is compatible with the chemical composition of the matrix used for calibration or used as input data in the application of numerical correction methods. In our case, the self-absorption corrections were carried out by using Gamma Vision software version 8 [39].

Finally, the Italian Accreditation Body (ACCREDIA) has formally acknowledged the elevated quality of HPGe analysis outcomes [41]. This implies the continuous verification (with an annual periodicity) of the maintenance of the performance characteristics of the gamma spectrometry method. In particular, the repeatability of the results was verified over time using the double test method. A certified reference material (also containing radionuclides ^40^K and ^137^Cs) was analysed twice, with the active concentration of the radionuclide of interest being defined as x_1_ (first measurement) and x_2_ (second measurement). The probability level *p* = 0.95 was considered. The following formula was used:

(2) $$\left|x_{1}-x_{2}\right|\leq \sqrt{2}\cdot s_{r}\cdot t$$

where t is the Student variable, and s_r_ is the standard deviation of repeatability obtained in the validation phase.

### 3.3. Radiological Health Risk

#### 3.3.1. Absorbed Gamma Dose Rate

The absorbed gamma dose rate, D (nGy h^−1^), for indoor exposure was determined according to [42]:

D = 0.92C_Ra_ + 1.1C_Th_ + 0.08C_K_ (3)

where C_Ra_, C_Th_, and C_K_ represent the mean specific activities (average value of the five analysed aliquots) of ^226^Ra, ^232^Th, and ^40^K in the analysed rocks, respectively.

#### 3.3.2. Annual Effective Dose Equivalent

To estimate the annual effective dose equivalent (AEDE) (mSv y^−1^), the following formula was applied, incorporating an 80% employment factor to account for indoor exposure on the basis of the standard room model reported in [42,43]:

AEDE = (D − 50) × 8760 h × 0.7 Sv Gy^−1^ × 0.8 × 10^−6^ (4)

This value should be below 1 mSv y^−1^ to ensure that the radiological risk is considered negligible [1,44].

#### 3.3.3. Activity Concentration Index

The European Commission has established an activity concentration index (ACI) to assess whether the dose criterion is met [42]:

ACI = (C_Ra_/300 + C_Th_/200 + C_K_/3000) (5)

This index is associated with the reference level for external exposure to gamma radiation released by building materials indoors (AEDE), in addition to outdoor exposure (i.e., 1 mSv y^−1^) [1,44]. As such, the ACI serves primarily as a tool to identify materials that may pose a risk in building applications, suggesting avoiding materials with I > 1 (i.e., corresponding to AEDE values higher than 1 mSv y^−1^).

#### 3.3.4. Alpha Index

The alpha index is calculated as [45]:

I_α_ = C_Ra_/200 (6)

This index measures the exposure to alpha radiation from indoor radon released by building materials. To minimise the exposure to radon indoors, the specific activity must be maintained below 200 Bq m^−3^. This can be achieved by ensuring that the activity concentration of ^226^Ra remains under 200 Bq kg^−1^. Consequently, for a very low risk of radiation exposure, the alpha index I_α_ must be less than 1.

### 3.4. ^222^Rn Exhalation Rate

**Figure 2:**
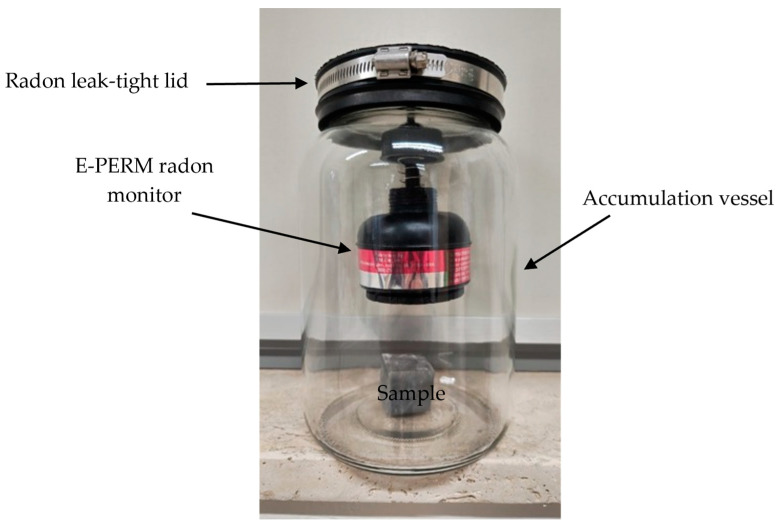
A schematic drawing of the experimental setup.

In order to determine the radon exhalation rate from each aliquot of the analysed natural stones, E-PERM electret ion chambers (Rad Elec Inc., Frederick, MD, USA) were used to measure the ^222^Rn activity that accumulated in the vessel after a specified build-up time. Temperature and humidity factors were controlled during the measurements. This method is referred to as the “accumulator method” [46] (see Figure 2).

The variation of ^222^Rn specific activity, C_Rn_ (Bq m^−3^), over time, in a sealed accumulation vessel, can be expressed as:

(7) $$C_{Rn}=\frac{E(1-e^{-\lambda_{Rn}t})m}{V_{A}\lambda_{Rn}}+C_{Rn}^{0}e^{-\lambda_{Rn}t}$$

where E is the specific exhalation rate (Bq kg^−1^ h^−1^) from the sample, λ_Rn_ is the decay constant for ^222^Rn (h^−1^), V_A_ is the volume of the accumulation vessel (m^3^), m is the sample mass (kg), and C^0^_Rn_ (Bq m^−3^) is the ^222^Rn activity concentration at the start of the accumulation time (t = 0) [47].

The E-PERM system functions by leveraging the ions generated during the radon decay process, thereby decreasing the electrical potential of a Teflon disk within a chamber that is charged electrostatically. Measurement of the radon concentration was accomplished through a combination of techniques and employing a 210-mL chamber configured with a short-term electret. Specifically, the radon activity concentration, C_Rn_ (Bq m^−3^), was determined by following the established relation:

(8) $$C_{Rn}=\frac{\left(V_{i}-V_{f}\right)}{C_{F}T}-BG$$

where V_i_ and V_f_ are the measured initial and final electret voltages, respectively; C_F_ is the calibration factor (V per Bq m^3^ d); T is the exposure interval (days); and BG is the background-related equivalent radon concentration of natural gamma radiation.

The calibration factor was calculated using:

(9) $$C_{F}=0.04589+0.0000155\frac{\left(V_{i}+V_{f}\right)}{2}$$

According to [48], the total volume for the E-PERM chamber, after subtracting the excluded volume, was found to be 3.8 L. It was therefore concluded that the “back diffusion” effect had no impact on the radon exhalation rate measurement, as the sample volume was more than ten times smaller than the chamber’s volume [49].

According to [45], the specific exhalation rate, E (Bq h^−1^ kg^−1^), is given by:

(10) $$E=\frac{(C_{Rn}V_{A}\lambda_{Rn})/m}{1-(1-e^{-\lambda_{Rn}t})/\lambda_{Rn}T}$$

In this study, the net ^222^Rn concentration was determined by subtracting the blank reading, which was obtained by placing E-PERM chambers into the empty jar utilised for the experiments and exposing it for the same duration as the experimental setup [49].

## 4. Results and Discussion

### 4.1. Radioactivity and Radon Exhalation Analysis

Table 1 reports the mean (averaged from the five aliquots of the analysed rocks), minimum, and maximum activity concentrations of ^226^Ra, ^232^Th, and ^40^K, for the investigated samples as well as the mean, minimum, and maximum specific ^222^Rn exhalation rate.

First of all, it is worth remarking that these findings are consistent with the natural radioactive levels observed in building materials used in Italy and elsewhere [50,51].

In particular, as far as the natural radioactivity content is concerned, it is important to point out that the specific activities of ^226^Ra and ^232^Th for both the investigated natural stones were lower than the world average values of 35 Bq kg^−1^ and 30 Bq kg^−1^, respectively, for this environmental matrix [1]. On the other hand, the specific activity of ^40^K exceeded the global average of 400 Bq kg^−1^ only for the sandstone rock. Taking into account that, as also frequently described in the literature, the activity concentrations of ^226^Ra, ^232^Th, and ^40^K are strictly dependent on the chemical composition and mineralogical phases of the samples under study [52], future investigations through analytical techniques such as X-ray fluorescence spectroscopy, micro-Raman scattering, and X-ray diffraction are planned in order to explore the chemical and mineralogical composition of these rocks in more detail, trying to clarify its role in the observed radioactivity levels [53,54,55].

As far as the radon exhalation rate from the analysed natural stones is concerned, it exhibited considerable variation, with values spread over several orders of magnitude. Of note, the mean values obtained aligned well with worldwide measurements [56,57,58].

Furthermore, the contribution of a specific stone to indoor radon accumulation can be estimated using the results of the radon exhalation measurements and the necessary schematic assumptions. The indoor radon concentration generated by the confining walls can be easily determined as follows:

(11) $$C_{\mathrm{Rn}}=\frac{EA_{r}}{V_{r}\lambda_{v}}$$

where A_r_ indicates the area of the wall surface; V_r_ is the volume of the confined space; and λ_v_ is the room ventilation rate. The ratio A_r_/V_r_ was 1.6 for a standard room of 4 × 5 × 2.8 m^3^, while the ventilation rate was assumed to be between 0.2 and 2 h^−1^ [59].

In this paper, the highest calculated mean value of E (i.e., 0.012 Bq h^−1^ kg^−1^ for the limestone rock) corresponded to an exhalation rate of about 0.17 Bq m^−2^ h^−1^. Assuming the lowest air ventilation rate (λ_v_ = 0.2), the resulting indoor radon concentration would be around 1.4 Bq m^−3^, thus considerably lower than the recommended limit of 300 Bq m^−3^ established by [44].

### 4.2. Evaluation of the Radiological Hazard Effects

The D, AEDE, ACI, and I_α_ for the investigated natural stones, as calculated by using Equations (3)–(6), are reported in Table 2.

It is noteworthy to highlight that D is related to the lithologic component of the sampling location, as extensively documented in the literature [60,61]. The gamma dose rates were found to be lower than the natural background value of 50 nGy h^−1^ and equal to 100 nGy h^−1^ for the limestone and sandstone rocks, respectively. Thus, the AEDE, due to the activities of ^226^Ra, ^232^Th, and ^40^K in the investigated samples, with the sandstone rock exhibiting a significant level of 247 µSv y^−1^, fell short of the established threshold value of 1 mSv y^−1^ in all cases [62], assuring a negligible radiation hazard to the public. The ACI was found to be 0.18 and 0.48 for the limestone rock and sandstone rock, respectively. Both these values were below 1, indicating negligible radiological risks from gamma radiation exposure [63]. Finally, concerning I_α_, it was found to be 0.16 and 0.06 for the limestone and sandstone rocks, respectively, which was always less than 1 even in this case [64,65].

## 5. Conclusions

The natural radioactivity content and radon exhalation of limestone and sandstone rocks from the archaeological park of Tindari in Sicily, southern Italy, materials used as construction materials in the area under investigation, were investigated by using HPGe gamma-ray spectrometry and E-PERM electret ion chambers, respectively, and reported as a case study.

Radiological parameters such as D, AEDE, ACI and I_α_, were all evaluated to assess any potential radiological hazards associated with radiation exposure from the analysed rocks. The results showed that the AEDE values for the analysed stones were below the action levels set by Italian legislation for population members, indicating no significant radiological risk. Furthermore, I and I_α_ were found to be less than unity, indicating a minor radiological risk from gamma radiation exposure and preventing the radon concentration exposure to indoor radon concentrations exceeding 200 Bq m^−3^, respectively. Simulations of indoor radon accumulation for a standard room constructed from limestone, which exhibited a higher radon exhalation rate, showed that even under poor ventilation conditions, the indoor radon concentration remained below the reference level of 300 Bq m^−3^, as established by Italian Legislation 101/20. This suggests that the use of these natural stones in dwellings and public buildings presents no substantial radiological risk.

## Figures and Tables

**Table 1:** Mean, minimum, and maximum activity concentrations of ^226^Ra, ^232^Th, and ^40^K for the analysed samples together with the mean, minimum, and maximum specific ^222^Rn exhalation rate (E).

Sample	Specific Activity			E (Bq h^−1^ kg^−1^)
	^226^Ra (Bq kg^−1^ d.w.)	^232^Th (Bq kg^−1^ d.w.)	^40^K (Bq kg^−1^ d.w.)	
Limestone rock				
Mean	32.8 ± 3.6	8.2 ± 1.4	96.2 ± 12.5	0.012 ± 0.003
Minimum	31.5 ± 3.2	7.5 ± 1.5	89.8 ± 13.1	0.011 ± 0.003
Maximum	33.9 ± 3.5	8.8 ± 1.3	103.8 ± 11.3	0.013 ± 0.002
Sandstone rock				
Mean	11.3 ± 1.6	26.9 ± 3.8	922 ± 110	0.005 ± 0.003
Minimum	10.1 ± 1.8	24.5 ± 3.9	856 ± 130	0.004 ± 0.002
Maximum	12.6 ± 1.5	28.6 ± 3.3	1011 ± 105	0.006 ± 0.003

**Table 2:** The D, AEDE, ACI, and Iα for the investigated natural stones.

Sample	D (nGy h^−1^)	AEDE (µSv y^−1^)	ACI	I_α_
Limestone rock	42.8	-	0.18	0.16
Sandstone rock	100	247	0.48	0.06

## Data Availability

Data are contained within the article.
